# Acute avulsion fracture of the ischial tuberosity in an adolescent athlete treated by suture anchor fixation using the suture bridge technique: a case report

**DOI:** 10.1093/jscr/rjae748

**Published:** 2024-11-25

**Authors:** Yasuyuki Omichi, Tomohiro Goto, Kaori Momota, Michihiro Takai, Ryosuke Sato, Tetsuya Enishi, Shunji Nakano, Koichi Sairyo

**Affiliations:** Department of Orthopedics, Tokushima Municipal Hospital, 2-34 Kitajousanjima, Tokushima, 770-0812, Japan; Department of Orthopedics, Tokushima Municipal Hospital, 2-34 Kitajousanjima, Tokushima, 770-0812, Japan; Department of Orthopedics, Tokushima Municipal Hospital, 2-34 Kitajousanjima, Tokushima, 770-0812, Japan; Department of Orthopedics, Tokushima Municipal Hospital, 2-34 Kitajousanjima, Tokushima, 770-0812, Japan; Department of Orthopedics, Tokushima Municipal Hospital, 2-34 Kitajousanjima, Tokushima, 770-0812, Japan; Department of Rehabilitation Medicine, Tokushima Municipal Hospital, 2-34 Kitajousanjima, Tokushima, 770-0812, Japan; Department of Orthopedics, Tokushima Municipal Hospital, 2-34 Kitajousanjima, Tokushima, 770-0812, Japan; Department of Orthopedics, Institute of Biomedical Sciences, Tokushima University Graduate School, 3-18-15 Kuramoto, Tokushima, 770-8503, Japan

**Keywords:** acute avulsion fracture of the ischial tuberosity, case report, longitudinal incision, subgluteal approach, suture anchor fixation, suture bridge technique

## Abstract

This is the first report of acute avulsion fracture of the ischial tuberosity (AFIT) treated by suture anchor fixation using the suture bridge technique. A 13-year-old boy developed sudden, severe right hip pain while running a short distance. Pelvic images revealed the avulsion fracture of the right ischial tuberosity with displacement of the avulsed fragment by 35 mm. We performed open reduction and reconstruction fixation 5 days after the injury using the subgluteal approach with longitudinal skin incision. Four suture anchors were set at the ischial tuberosity, and the avulsed fragment was repositioned and fixed using the suture bridge technique. At 1 year postoperatively, the avulsion fracture was bony fused, and he had returned to his preinjury competitive level. Use of multiple suture anchors increases the strength of fixation, which overcomes the problem of anchor loosening and makes open reduction and reconstruction fixation an effective treatment for acute avulsion fracture of the ischial tuberosity.

## Introduction

Apophyseal avulsion fractures of the pelvis typically occur in adolescent athletes. Avulsion fractures of the ischial tuberosity (AFIT) account for 10%–30% of these fractures [1]. Surgery is recommended for severely displaced avulsed fragments [2]. Open reduction and internal fixation (ORIF) using screws or plates is usually performed for acute AFIT with displacement [2]. However, there are few reports of ORIF for avulsion fractures being performed with suture anchors using the suture bridge technique [3, 4].

This report is the first to describe a case of acute AFIT treated by suture anchor fixation using the suture bridge technique.

## Case report

Informed consent was obtained from the patient and his parents for publication of his clinical information. The patient was a 13-year-old Japanese boy who presented to the emergency department with severe right hip pain and inability to walk. He was a track and field athlete, specializing in short-distance running, and had developed sudden severe right hip pain while running a short distance. He had a height of 168 cm, a weight of 49 kg, and a body mass index of 17.4 kg/m^2^.

**Figure 1:**
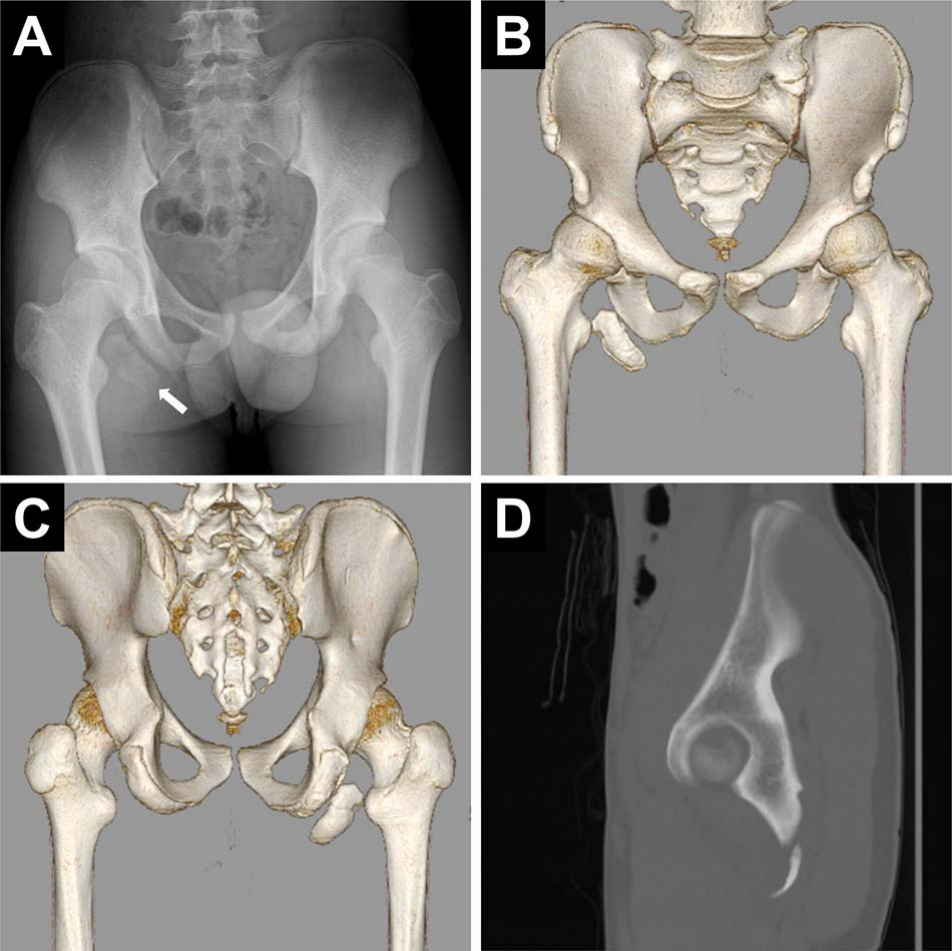
Preoperative images of the pelvis. (A) Pelvic radiograph showing the displaced avulsion fracture of the ischial tuberosity (white arrow). (B, C) Computed tomography scans show the displaced avulsed fragment. The avulsed fragment was displaced by 35 mm. (D) The size of the avulsed fragment was 55 × 17 mm.

**Figure 2:**
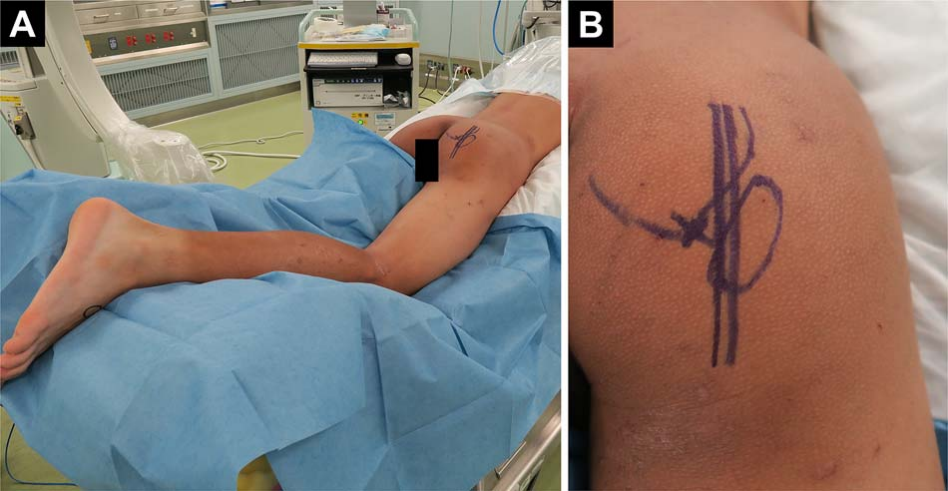
Patient positioning and skin incision. (A) The patient in the prone position with the hip and knee joints in slight flexion after general anesthesia. (B) Longitudinal skin incision 1 cm lateral to the ischilal tuberosity.

**Figure 3:**
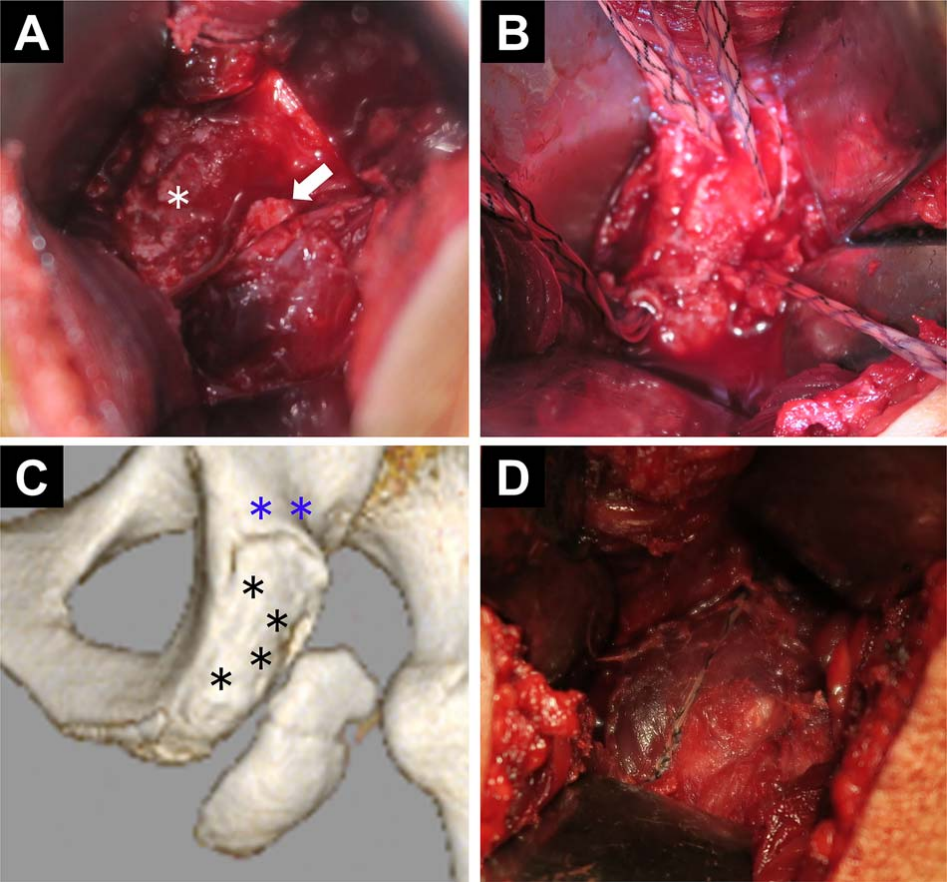
Intraoperative findings and diagram. (A) The avulsed fragment is displaced in the distal direction (white arrow). The asterisk indicates the ischial tuberosity. (B) Four suture anchors at the ischial tuberosity. (C) Diagram with computed tomography images. The blue asterisks indicate the two holes proximal to the ischial tuberosity used to fix the anchor tapes. The black asterisks indicate the four suture anchor holes at the ischial tuberosity. (D) Fixation of the avulsed fragment by suture anchors using the suture bridge technique.

Pelvic images revealed the avulsion fracture of the right ischial tuberosity with severe displacement of the avulsed fragment (Fig. 1A–D). We performed ORIF 5 days after the injury. The patient was placed in the prone position under general anesthesia with the hip and knee joints in slight flexion (Fig. 2A). A 10-cm longitudinal skin incision was made 1 cm lateral to the ischial tuberosity and the subgluteal approach was performed (Fig. 2B). The avulsed fragment was displaced in the distal direction (Fig. 3A) and repositioned by posterior extension of the hip and flexion of the knee joint.

**Figure 4:**
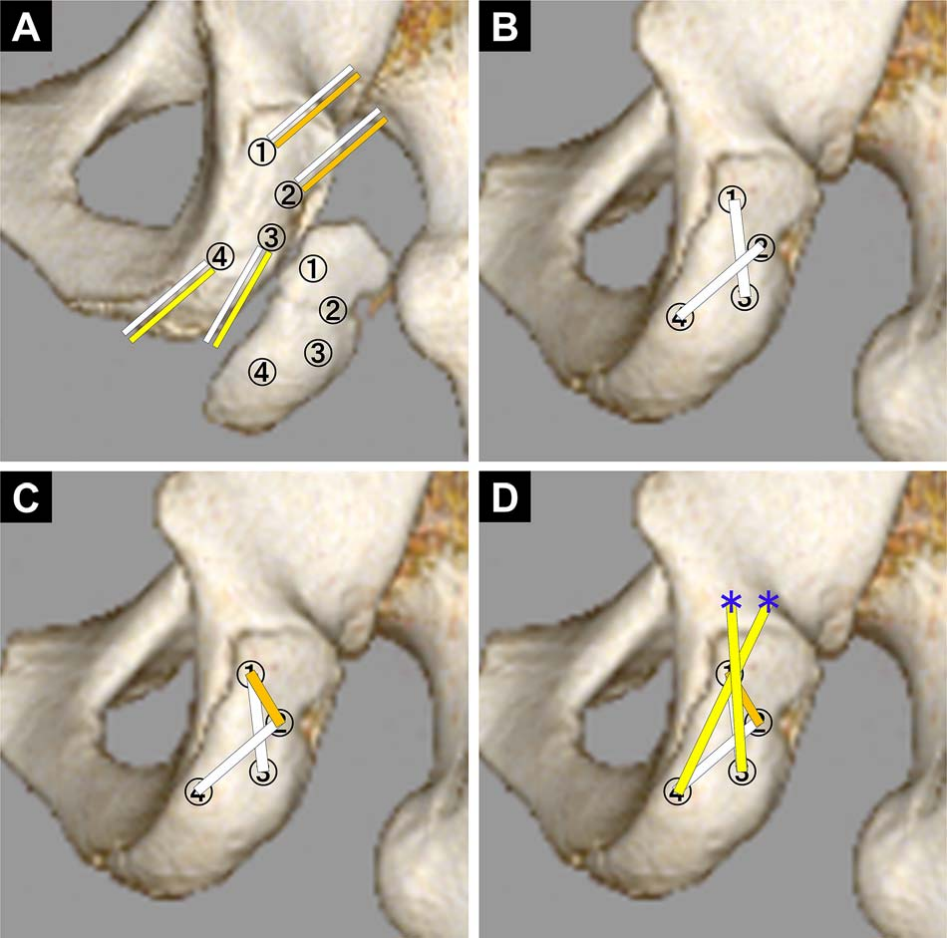
Diagram of the pattern of how to pass the suture tapes. (A) First. Two suture tapes connected to each anchor of the ischilal tuberosity were passed through each of the same number of bone holes in the avulsed fragment. (B) Second. With the avulsed fragment repositioned, the suture tapes No. 1 and No. 3, and No. 2 and No. 4 were tied together to fix the avulsed fragment (white line). (C) Third. The suture tapes No. 1 and No. 2 were tied (orange line). (D) Final. The suture tapes No. 3 and No. 4 were crossed and fixed with absorbable screws (yellow line: suture tape, blue asterisk: absorbable screw to fix the suture tape).

Four suture anchors were set at the ischial tuberosity (Biocomposite-Corkscrew 5.5 mm with suture tape, Arthrex, Naples, FL) (Fig. 3B). Next, considering the location of the anchors, four bone holes were created in the avulsed fragment with 1.8-mm Kirschner wire. Two holes were drilled slightly proximal to the ischial tuberosity to fix the suture tapes (Suture Anchor, DX Knotless SwiveLock, Biocomposite-Corkscrew 4.75 mm, Arthrex) (Fig. 3C). The suture tapes connected to the anchors were passed through the four bone holes in the avulsed fragment, which was repositioned and fixed using the suture bridge technique (Figs 3D and 4A–D).

Postoperatively, the range of motion (ROM) of the knee brace was set at 60°–120° for the first 3 weeks and with no restrictions from 6 weeks. Passive ROM training was started postoperatively within the ROM set by the brace. Active ROM training was started after 6 weeks. One-third weight-bearing was permitted from 6 weeks, and full weight-bearing was allowed from 9 weeks onwards. Jogging was started 3 months postoperatively, as well as core and hamstring muscle strengthening exercises. The patient returned to competitive play 5 months after surgery.

**Figure 5:**
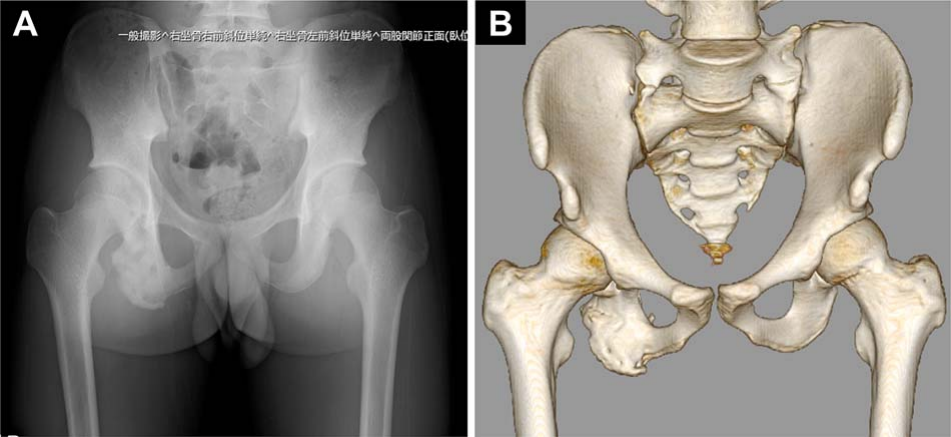
Postoperative images of the pelvis. (A) Plain radiograph. (B) Computed tomography scan.

His hamstring strength measured using a hand-held dynamometer recovered to 80% of that on the unaffected side by 6 months after surgery and was maintained thereafter (Table 1). Pelvic images showed bony fusion of the avulsed fragment (Fig. 5). One year after the surgery, he returned to his preinjury competitive level and broke his own record for short-distance running. His Perth Hamstring Assessment Tool [5] score was 100 points (100%).

**Table 1:** Postoperative right and left hamstring strength at 90° of knee flexion as measured using a hand-held dynamometer.

Months after surgery	Hamstring strength		
	Right (nm/kg)	Left (nm/kg)	Right/left
4	0.46	1.00	60%
6	0.98	1.21	80.9%
8	1.13	1.27	89.1%
10	0.93	1.12	83%
12	0.90	1.08	83%

## Discussion

There is still no guideline for treatment of AFIT, but a conservative approach is generally accepted when an avulsed fragment is not severely displaced [1]. If the displacement is severe, non-union, residual pain, inability to return to sports, and chronic sciatic nerve symptoms often occur [1, 2]. Recent reports recommend surgical treatment when the avulsed fragment is displaced by more than 1.5 cm [2, 6]. Early diagnosis and appropriate treatment for AFIT are important.

There are several surgical options for acute AFIT, including reconstruction plates, cancellous screws, and cortical screws [7, 8]. Suture anchors are an alternative to these treatments. In recent years, there have been some reports of ORIF using the suture bridge technique for chronic AFIT [9, 10]. In a cadaveric study, Hamming *et al.* examined the strength of the anchors used for reconstruction of the hamstring tendons and found that repair using five anchors was significantly stronger than that using two anchors [11]. We have obtained good results with four suture anchors, which is consistent with the previous reports. When using suture anchors for AFIT, the strength of fixation can be increased by placing more anchors.

There have been some recent reports on functional outcomes and return to sports following surgical procedures for AFIT [1, 12]. Best *et al.* [1] found that 90% of 10 adolescent athletes with AFIT treated with suture anchors returned to sports. In a study by Williams *et al.* [12], 16 adolescent athletes with AFIT were treated using screws, suture anchors, cortical suspensory buttons, and screws with anchors. Williams *et al.* [12] reported that all patients returned to sports, and they concluded that the functional outcome was not affected by the fixation method used. In addition, good results and high satisfaction can be expected in cases with a displaced AFIT regardless of the fixation method used [2]. In this regard, the suture anchor method is a useful option.

In our case, we performed the subgluteal approach with a longitudinal skin incision. Generally, a transverse skin incision is made parallel to skin creases when the subgluteal approach is performed. However, AFIT is rare, and most orthopedic surgeons have never experienced the subgluteal approach. Sciatic nerve injury is one of the serious complications of the subgluteal approach and can be prevented by ensuring a secure surgical field and operating with caution. The subgluteal approach with a longitudinal skin incision provides a wide surgical field and can be performed safely by surgeons with limited experience of using this approach.

In conclusion, this is the first report of ORIF with four suture anchors using the suture bridge technique for acute AFIT. Use of multiple suture anchors increases the strength of fixation, which overcomes the problem of anchor loosening and makes ORIF an effective treatment for acute AFIT.

## Data Availability

The datasets used and/or analyzed during the current study are available from the corresponding author on reasonable request.

## References

[1] Best R , Meister A, Huth J, et al. Surgical repair techniques, functional outcome, and return to sports after apophyseal avulsion fractures of the ischial tuberosity in adolescents. Int Orthop 2021;45:1853–61. 10.1007/s00264-021-04959-w. 33963885 PMC8266717

[2] Nauta HJA , van der Made AD, Tol JL, et al. Satisfactory clinical outcome of operative and non-operative treatment of avulsion fracture of the hamstring origin with treatment selection based on extent of displacement: a systematic review. Knee Surg Sports Traumatol Arthrosc 2021;29:1813–21. 10.1007/s00167-020-06222-y. 32809117 PMC8126544

[3] Furuhata R , Kamata Y, Kono A, et al. Surgical repair using suture bridge technique for triceps tendon avulsion. Case Rep Orthop 2021;2021:5572126. 10.1155/2021/5572126. 33968456 PMC8081627

[4] Kim KS , Suh DW, Park SE, et al. Suture anchor fixation of comminuted inferior pole patella fracture-novel technique: suture bridge anchor fixation technique. Arch Orthop Trauma Surg 2021;141:1889–97. 10.1007/s00402-020-03671-5. 33125547

[5] Blakeney WG , Zilko SR, Edmonston SJ, et al. Proximal hamstring tendon avulsion surgery: evaluation of the Perth hamstring assessment tool. Knee Surg Sports Traumatol Arthrosc 2017;25:1936–42. 10.1007/s00167-016-4214-y. 27344550

[6] Eberbach H , Hohloch L, Feucht MJ, et al. Operative versus conservative treatment of apophyseal avulsion fractures of the pelvis in the adolescents: a systematical review with meta-analysis of clinical outcome and return to sports. BMC Musculoskelet Disord 2017;18:162. 10.1186/s12891-017-1527-z. 28420360 PMC5395880

[7] Kaneyama S , Yoshida K, Matsushima S, et al. A surgical approach for an avulsion fracture of the ischial tuberosity: a case report. J Orthop Trauma 2006;20:363–5. 10.1097/00005131-200605000-00012. 16766942

[8] Sezer HB , Hardy A, Bohu Y, et al. Treatment of acute bony avulsion of ischial tuberosity with cortical screw fixation. Arthrosc Tech 2021;10:e2691–8. 10.1016/j.eats.2021.08.011. 35004150 PMC8719112

[9] Biedert RM . Surgical management of traumatic avulsion of the ischial tuberosity in young athletes. Clin J Sport Med 2015;25:67–72. 10.1097/JSM.0000000000000088. 24662573

[10] Tetsunaga T , Endo H, Tetsunaga T, et al. Avulsion fracture of the ischial tuberosity treated with the suture bridge technique: a case report. BMC Musculoskelet Disord 2019;20:9. 10.1186/s12891-018-2377-z. 30611250 PMC6320617

[11] Hamming MG , Philippon MJ, Rasmussen MT, et al. Structural properties of the intact proximal hamstring origin and evaluation of varying avulsion repair techniques: an in vitro biomechanical analysis. Am J Sports Med 2015;43:721–8. 10.1177/0363546514560878. 25527082

[12] Williams BA , Titus M, Chaclas N, et al. Surgically treated ischial tuberosity avulsion fractures in adolescents: risks and outcomes of 3 fixation constructs. J Pediatr Orthop 2024. Online ahead of print. 10.1097/BPO.0000000000002799. 39188122

